# Identification of Rays through DNA Barcoding: An Application for Ecologists

**DOI:** 10.1371/journal.pone.0036479

**Published:** 2012-06-11

**Authors:** Florencia Cerutti-Pereyra, Mark G. Meekan, Nu-Wei V. Wei, Owen O'Shea, Corey J. A. Bradshaw, Chris M. Austin

**Affiliations:** 1 Research Institute of Environment and Livelihood, Charles Darwin University, Northern Territory, Australia; 2 Australian Institute of Marine Science, UWA Oceans Institute, Western Australia, Australia; 3 Murdoch University, Western Australia, Australia; 4 The Environment Institute and School of Earth and Environmental Sciences, The University of Adelaide, Adelaide, South Australia, Australia; 5 South Australian Research and Development Institute, South Australia, Australia; 6 School of Science Monash University Sunway Campus, Petaling Jaya, Selangor, Malaysia; Barnard College, Columbia University, United States of America

## Abstract

DNA barcoding potentially offers scientists who are not expert taxonomists a powerful tool to support the accuracy of field studies involving taxa that are diverse and difficult to identify. The taxonomy of rays has received reasonable attention in Australia, although the fauna in remote locations such as Ningaloo Reef, Western Australia is poorly studied and the identification of some species in the field is problematic. Here, we report an application of DNA-barcoding to the identification of 16 species (from 10 genera) of tropical rays as part of an ecological study. Analysis of the dataset combined across all samples grouped sequences into clearly defined operational taxonomic units, with two conspicuous exceptions: the *Neotrygon kuhlii* species complex and the *Aetobatus* species complex. In the field, the group that presented the most difficulties for identification was the spotted whiptail rays, referred to as the *‘uarnak’* complex. Two sets of problems limited the successful application of DNA barcoding: (1) the presence of cryptic species, species complexes with unresolved taxonomic status and intra-specific geographical variation, and (2) insufficient numbers of entries in online databases that have been verified taxonomically, and the presence of lodged sequences in databases with inconsistent names. Nevertheless, we demonstrate the potential of the DNA barcoding approach to confirm field identifications and to highlight species complexes where taxonomic uncertainty might confound ecological data.

## Introduction

Taxonomic misidentification and the presence of cryptic species can seriously compromise the veracity of ecological, fisheries and conservation-related research and management [1]–[4]. These problems are further compounded by the ‘greying’ of the taxonomic workforce and the decline in the teaching of taxonomy and training of field biologists at universities, both issues identified as major impediments to the conduct of biodiversity science and conservation biology [5]. Within this context, a key question is: how much confidence can be placed in the application of correct scientific names of taxa reported in ecological studies? In a review of high-ranking ecological journals, Bortolus [4] reported that 62.5% of papers did not provide any supporting information justifying or guaranteeing the correct identification of the organisms under investigation.

The challenges for ecologists seeking verification of their field-based identifications are not trivial. Even when adequate taxonomic keys and field guides are available, it is often difficult to identify organisms in the field with confidence, as ecologists can be dealing with juveniles, undocumented geographic variants, or sexual dimorphism, such that accurate identification might require examination of microanatomy or measurements of a complex combination of morphometric attributes. Handling, examining and measuring individuals is often impractical, inappropriate for ethical reasons, or simply dangerous, thus exacerbating the problem of securing accurate identification. Furthermore, even if experienced taxonomists have studied the target organisms, it is unlikely that they can be encouraged to assist in the field, especially in remote locations. Voucher specimens can be taken for subsequent lodgement in museums; however, this is often impractical for large species, samples obtained in remote locations and studies involving multiple species. Even where voucher specimens can be obtained, it will not necessarily guarantee reliable and timely identification.

DNA barcoding potentially offers scientists who are not expert taxonomists a powerful tool to support the efficiency and accuracy of field studies involving the challenging identification of diverse taxa [6]. The proponents of this approach mostly advocate the use of a single gene for global identification of animals based on the availability of a library of sequences linked to voucher specimens, thus making these sequences, in effect, a DNA barcode [7], [8]. A 650-base fragment of the cytochrome *c* oxidase I (COI, *cox1*) is proposed as a ‘global’ standard because the variation in COI within species is lower relative to that among species. While the DNA barcoding approach has its critics when touted as a solution to impediments presented by traditional taxonomy [9], [10], it does potentially provide a quick and reliable means to confirm the identification of individuals in the field and to identify groups where there is discordance in the delineation of species boundaries that require further research. In their paper on DNA Barcoding Australian chondrichthyans, Ward *et al*., [6] recommend this approach for marine ecologists working on chondrichthyans in the absence of expert taxonomists.

While the taxonomy of rays has received reasonable attention in some parts of the world, including Australia [11] where DNA information is accumulating, the fauna in remote locations such as Ningaloo Reef, Western Australia remains relatively poorly studied. It is now becoming apparent that the field identification of some species without access to taxonomic expertise or the ability to evaluate diagnostic traits (e.g. morphometrics or microanatomy) is problematic. Recent studies indicate that morphologically cryptic elasmobranchs might be common, as some groups show ontogenetic colour variation and colour pattern similarities among different species [12]–[15]. For example, a recent revision of the ‘whiptail ray complex’ found that coloration patterns changed with life stage and different habitats [16], thus complicating field identification.

Here, we report an application of DNA-barcoding to confirm the identification of rays as part of ecological studies at Ningaloo Reef. The establishment of the Ningaloo Reef Ecosystem Tracking Array (NRETA), which is part of the Australian Animal Tagging and Monitoring System (AATAMS, www.imos.org.au/aatams.html), a national network of acoustic stations, provided the opportunity to address the lack of knowledge of the spatial ecology of these animals by enabling a study of the fine-scale movement of a diverse community of rays inhabiting this reef system (Cerutti-Pereyra et al. unpublished data) In these studies, 70 individual rays including both juveniles and adults representing 17 presumed species were captured and fitted with acoustic tags and monitored for more than two years. Tissue samples were taken from each tagged individual for DNA barcoding. We therefore present 67 new COI sequences from these 17 putative species of rays to confirm field identification based on sequences deposited in the GenBank database. Our over-arching aim was to assess the potential of DNA barcoding as an aid to batoid species identification for the tagging study.

## Methods

### Study group

Rays, or batoids, include a variety of fishes closely related to sharks. Recent immunological and molecular studies show an ancient split between the two groups, where batoids are a sister group to the clade consisting of all shark orders [17]–[19]. Even though the monophyly of batoids is widely accepted, interrelationships within batoids remain controversial. Although early research established six orders, recent work now recognizes five: electric rays (Torpediniformes), skates (Rajiformes), guitar fishes (Rhinobatiformes), sawfishes (Pristioformes), and stingrays (Myliobatiformes) [20], [21]. Worldwide, there are between 507 and 630 species, many of them poorly known and requiring further taxonomic studies. Recent molecular evidence focuses on relationships among elasmobranch orders, but few studies have addressed interrelationships within the rays, e.g. [21], [22], [23].

The central Indo-Pacific is a major centre of origin and radiation of stingrays [19] and within this region, the Indo-Australian archipelago contains 30% of all species of sharks and rays worldwide [11], [24], including many species of tropical rays. Rays are exploited directly or indirectly in commercial fisheries; however, detailed data on landings and by-catch are often lacking. Global reviews of batoid fisheries indicate that in most cases there are large gaps in the basic biological information required to implement strategic management plans for stocks [25], [26] and over-fishing has been suggested to be one of the critical reasons for the decline and local extinction of populations of rays and sharks in both hemispheres [26]–[32].

DNA information for species of rays is accumulating, including COI sequences with 1255 lodged on GenBank to date. This suggests that there is now a sufficient DNA database available to at least partially support a DNA barcoding approach for taxonomic identification of batoids.

### Study site

Ningaloo Reef is the largest fringing reef system in the Southern Hemisphere and extends along 270 km of coastline in the north of Western Australia. The reef is separated from the coast by a 0.2 to 7 km wide sandy lagoon, which is backed by a dry coastal plain [33], [34].

### Sampling

We used gill and hand nets, hook and line, a Hawaiian sling with a modified tip [35], and indigenous spear fishing to obtain tissue samples of rays. These were stored in a salt-saturated dimethyl sulphoxide (DMSO) solution (20% DMSO, 0.25 M EDTA, saturated with NaCl) in the field, then at −80°C in the laboratory. We visually identified and took disc-width measurements of each animal during handling or prior to taking tissue samples in the case of free-swimming rays.

We collected tissue samples from two individuals per species per site where possible. We also obtained samples from the Northern Territory, Lizard Island (Queensland), and Ha Long Bay (Vietnam) for comparison. The samples were collected by different researchers and fishermen; when possible, a provisional identification was made in the field. The individual samples, their geographic origin and initial taxonomic identification based on information provided by Last and Stevens [11], are shown in Table S1.

### Laboratory procedures

We extracted genomic DNA from muscle tissue using DNeasy Blood & Tissue Kit, and amplified the COI gene by polymerase chain reaction (PCR) using the universal primers FishF2 (5′TCGACTAATCATAAAGATATCGGCAC3′) and FishR2 (5′ACTTCAGGGTGACCGAAGAATCAGAA3′) designed by Ward *et al.* [6]. Each 50 µl reaction contained 5 µl of DNA tissue (ca. 10 ng), 4 µl (0.2 mM) of total Bioline dNTPs, 3 µl (0.6 µM) of each primer, 0.1 µl of 5 U/µl Mango taq, 5 µl of 10x Mango buffer, and 2 µl (2 mM) of MgCl_2._ PCR cycle conditions were an initial 3 min denaturation at 94°C, followed by 35 cycles of 50 sec at 94°C, 2 min at 50°C, 1.5 min at 72°C and finished with 6 min at 72°C. We examined the PCR products on 1% agarose gels, purified with QIAGEN QIAquick PCR Purification kit and sequenced with the automated sequenced using the dye-termination method (BigDye Terminator v3.1, Applied Biosystems). We sequenced amplicons in both forward and reverse directions. Chromatograms were inspected for noisy and ambiguous base calling and translated to check for stop codons. Noisy tails were trimmed. Only those consisting of more than 519 bp were used for the analysis. Several sequences trimmed to less than 519 bp were excluded from the phylogenetic analysis but were submitted to online databases for identification. Sequences used for the phylogenetic analysis were submitted to GenBank database under the accession numbers given in Table S1.

### Analysis

We assembled the sequence data using Mesquite 2.74 and revised our identification of samples after considering the results of two analyses. First, we submitted the sequences one at a time to the BOLD Identification Engine (www.boldsystems.org) and GenBank nucleotide database (www.ncbi.nlm.nih.gov/nucloetide). Both engines matched each uploaded sequence with every other sequence present in their databases and provided a percentage similarity with matching sequences (Table S2). In the second analysis, we constructed phylogenetic trees using ray sequences downloaded from both the GenBank nucleotide database and BOLD identification engine (Table S3). We chose sequences from GenBank/BOLD on the basis that they represented either the same species, a congeneric species, or they showed a high similarity to our sequences submitted to a blast search in GenBank or BOLD engines. If a species on GenBank displayed multiple divergent haplotypes, we chose sequences to represent this variation. We assembled these sequences with ours and aligned them using MEGA 4 [36].

The data set used for phylogenetic analysis was composed of only those sequences that consisted of a minimum of 519 bp after trimming. We used both neighbour-joining (NJ) and Bayesian methods of phylogenetic tree construction for analysis. Neighbour-joining has a strong track record of being able to rapidly analyze large datasets [37]. Modeltest 3.7 showed that the Hasegawa, Kishino and Yano [38] (HKY85) model of molecular evolution was the most appropriate for our dataset [39]. However, we also used the simple Kimura two-parameter model to estimate genetic distance [40] as it is the standard model of molecular evolution used in barcoding studies [41]. We used sequences from two species of sharks (*Carcharhinus amblyrhyncos* Bleeker, 1856 and *C. plumbeus* Nardo, 1827) and two species of rays (*Pristis clavata* Garman 1906 and *Torpedo californica* Ayres, 1855) from GenBank as outgroups in separate analyses. As the relationships at the level required for species discrimination did not change with the use of different outgroups, we only present the trees using shark taxa because we can be certain that these are an outgroup rather than an ingroup for batoids. We constructed trees using both nucleotide models with PAUP* 4.0b10 [42] and MrBayes [43]. As these provided similar outcomes, we only present results based on the neighbour-joining tree using the Kimura two-parameter model with bootstrap values and posterior probabilities.

**Figure 1:**
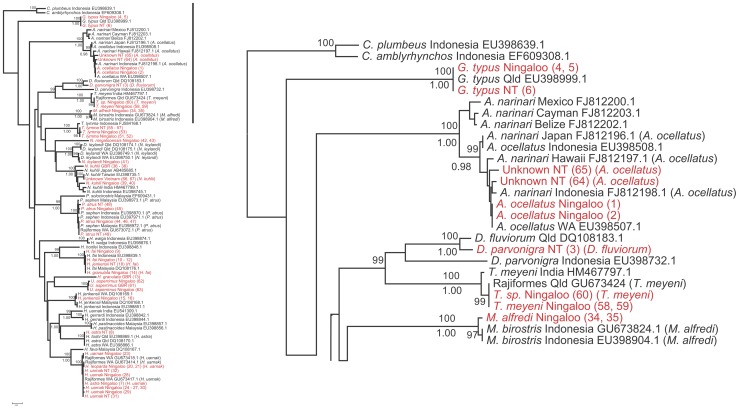
Phylogenetic relationship of rays Part I. Reduced view of the neighbour-joining tree based on COI sequence data using Kimura-two-parameter substitution model (left); the first part of the tree (right). Names in red are the sequences obtained in this study, the corrected nomenclature is in () and given in Table S2.

**Figure 2:**
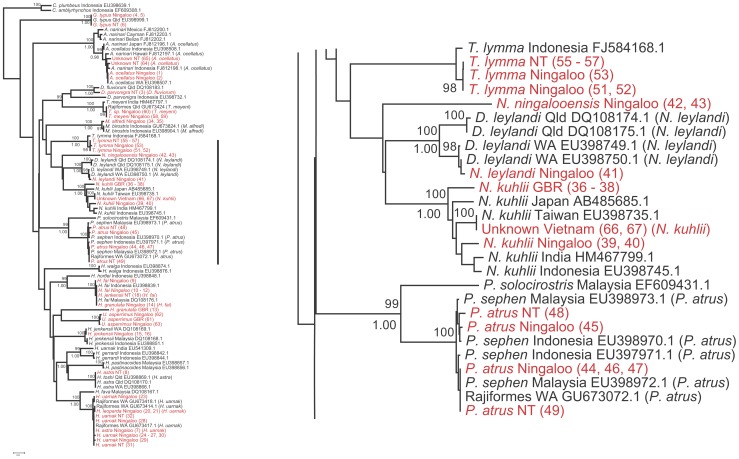
Phylogenetic relationship of rays Part II. Second part of the Neighbour-joining tree based on COI sequence data using Kimura-two-parameter substitution model (left); the second part of the tree (right). Names in red are the sequences obtained in this study, the corrected nomenclature is in () and given in Table S2.

**Figure 3:**
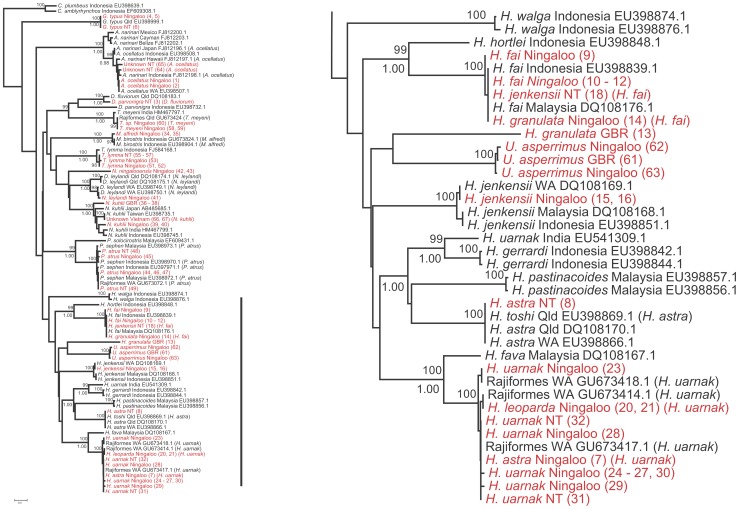
Phylogenetic relationship of rays Part III. Third part of the Neighbour-joining tree based on COI sequence data using Kimura-two-parameter substitution model (left); the third part of the tree (right). Names in red are the sequences obtained in this study, the corrected nomenclature is in () and given in Table S2.

We generated uncorrected pair-wise distances in PAUP* 4.0b10 [42], updating the name of the sequences used as detailed in Table S2. For initial species delineation, we grouped individuals that clustered with similarity <3.5% of divergence, which is the threshold recommended for COI of marine fish [6], [44]–[46] and equates to approximately 10x the intra-species variation proposed by Hebert *et al.,* [8]. We also used multi-dimensional scaling (MDS) in SPSS to explore patterns of variation in groups displaying high intra-speciation or geographic variation. For ease of interpretation and readability, we present the neighbour-joining tree divided into 3 sections (Figures 1, 2, and 3).

## Results

### General findings

We barcoded 67 individuals representing 17 putative ray species and five unidentified individuals for a fragment of the COI gene with an average length of ∼550 base pairs. When translated all sequences showed no stop codons, indication of heteroplasmy or NUMTs. All 67 sequences were compared with those in BOLD and GenBank databases (Table S2) to confirm the initial identification. Sixty one individuals with a minimum of 519 bp were included in the phylogenetic analysis verified by forward and reverse primers. Six sequences of less than 519 bp were excluded from this analysis. A neighbour-joining tree (Figures 1, 2, and 3) summarizes the relationships among samples from our study and matching sequences from the same or related species available on both nucleotide databases. After comparisons of our sequences with those on BOLD and GenBank databases, we present data for 16 species belonging to 10 genera, 3 families and 2 orders.

We barcoded 20 rays tagged as part of an ecological study at Ningaloo Reef (Cerutti-Pereyra et**al. unpublished data) to confirm or correct field identifications (Table S2). Sequences of *Himantura uarnak*, *H. fai, H. granulata, Aetobatus ocellatus*, *Pastinachus atrus, Taeniurops meyeni, Manta alfredi, Taeniura lymma,* and *Urogymnus asperrimus* represent new sequences from Australia for the GenBank nucleotide database. Data for *M. alfredi,* and *P. atrus* represent new sequences from Australia for both BOLD and GenBank databases. Sequences for *Neotrygon ningalooensis* have no matching sequences in either the GenBank or BOLD databases and new sequences of *N. kuhlii* from Vietnam are also presented.

The average congeneric distance (D = 8.5%) was 14 times the average conspecific distance (D = 0.63%) (Table 1). These calculations excluded the aberrant samples *D. parvonigra* from Indonesia (D = 9%) (GenBank accession number EU398732) and *H. uarnak* from India (D = 12%) (GenBank accession number EU541309.1). Approximately 90% of within-species values had <2% divergence; ∼20% of these had <1% divergence and 10% had between 2 and 3% divergence.

**Table 1:** Means and ranges of K2P distance values (%) for the COI gene region at different taxonomic levels for the ray species analysed in this study.

Comparisons	No. of comparisons	Mean	Minimum	Maximum
Between individuals within species	60	0.63	0	3.00
Between species within genera	20	8.85	3.40	14.00

### Taxonomic identification and barcoding

The COI sequences for the combined dataset (Figures 1, 2, and 3) grouped sequences into clearly defined operational taxonomic units, with two conspicuous exceptions. These consisted of what we refer to as *N. kuhlii* and *Aetobatus* species complexes. Spotted whip-tail rays presented the most difficulties for field identification and are referred to as the *‘uarnak’* complex. Complete consistency in field identification (often by different researchers) and the nomenclature of records held on GenBank and BOLD occurred for only one species, *T. lymma*, although the tree suggests a phylogeographic disjunction between the Indonesian and Australian samples of this species. Sequences of the recently described species *Neotrygon ningalooensis* [47] were placed in the same lineage with *N. leylandi* and *N. kuhlii* in the tree, but formed a clear and isolated cluster with an average genetic distance of 9% compared with other species within this genus.

Of the 67 sequences we tested, only 19 had consistent matches on both BOLD and GenBank (Table S2). As a consequence, there were a number of anomalies that meant that taxonomic identification was not straightforward or consistent. These anomalies were due to the presence of cryptic species, misidentification of species associated with sequences in the databases, or field misidentification of species in this study. We discuss these taxonomically complex groups and anomalies (Tables S1, S2) in more detail below:

#### *Manta birostris* (Walbaum, 1792)/*Manta alfredi* (Krefft, 1868) [48]

The submission of sequences identified as *M. alfredi* (# 34, 35) in both online databases produced matches of 99–100% with *M. birostris.* Sequences of this species showed a phylogeographic disjunction in the NJ tree between the Indonesian and Australian samples in the tree, but a genetic divergence of <1%.

#### *Urogymnus asperrimus* (Bloch & Schneider, 1801)

Sequences of this species from Ningaloo Reef (# 62, 63) and the Great Barrier Reef (# 61) clustered together in the tree and had an average genetic divergence of 0.32%. The submission of our sequences to GenBank produced either incorrect matches or matches only to the level of order (Table S2). Our sequences had matches of 98–100% in the BOLD database for *U. asperrimus*.

#### Glaucostegus typus (Bennet, 1830)

Sequences from Queensland (EU398732.1), Northern Territory (# 6), and Western Australia (#4, 5) were identical. Our sequences in GenBank had 99–100% similarity with *G. typus* and 100% similarity in BOLD with *G. typus* and *Rhinobatos typus* (senior synonym of *Glaucostegus*).

#### *Dasyatis parvonigra* (Last & White, 2008) [49]

A single specimen identified as *D. parvonigra* from Shoal Bay, Northern Territory (# 3) had a 98% similarity with a sequence on GenBank labelled as *D. fluviorum* (GenBank accession number DQ108183.1) from New South Wales, Australia and a 99% similarity with a sequence on BOLD labelled as *Dasyatis* sp. from Indonesia. Furthermore, a sequence from Indonesia recorded as *D. parvonigra* (EU398732.1), while placed in the same lineage, differed by 9%, whereas the average divergence with *D. fluviorum* from New South Wales was 1.6%.

#### *Pastinachus sephen* (Forsskal 1775)/*Pastinachus atrus* (Macleay, 1883)

Six sequences from rays identified by different researchers as *P. atrus* from Ningaloo Reef (# 44–47) and the Northern Territory (# 48, 49) clustered tightly with samples of *P. sephen* from Malaysia and Indonesia. The average genetic distance among samples was 0.29%. In GenBank, the most closely matched sequences were labelled *P. sephen*. In BOLD the highest matches (100%) included sequences identified as both *P. atrus and P. sephen*.

#### *Taeniurops meyeni* (Muller & Henle, 1841)

Of the three *Taeniurops* rays sampled from Ningaloo Reef, two were initially identified as *Taeniurops meyeni* [11] (# 58, 59) whereas the other was thought possibly to represent a new species because of an unusual colour pattern. The latter was provisionally referred to as *Taeniurops* sp (# 60). These sequences from Ningaloo Reef (*n* = 3) and one sequence under the name of *Rajiformes* (GenBank accession number GU673424.1) from Queensland were clustered tightly in the tree. There was a small difference between the Australian cluster and the sequence from India; however, the genetic distance among these sequences was low (0.36%). The matching entries in both GenBank and BOLD were labelled as *Taeniura meyeni.* Last and Stevens [11] revised the nomenclature of this species from *Taeniura to Taeniurops.*

#### *Neotrygon leylandi* (Last & White 2008) [50]

Sequences from Western Australia (*n* = 4) and Queensland (*n* = 3) for this species showed geographic variation with an average genetic distance among groups of 3% compared to 0.13% within groups. Our sequence from Ningaloo Reef, W.A. (# 41), matched 100% with sequences in BOLD labelled as *N. leylandi* and 99% with sequences in GenBank labelled *Dasyatis leylandi* (Last 1987) [51] (senior synonym of *Neotrygon*).

#### *Himantura fai*, Jordan & Seale, 1906/*H. jenkinsii* (Annandale, 1909)

Three samples (# 15–17) from Ningaloo Reef identified in the field as *H. jenkinsii* matched sequences (99–100%) in both GenBank and BOLD. However, a different sample from the Northern Territory (# 18) also initially identified as *H. jenkinsii* matched a different species in GenBank (*H. fai*) and both *Himantura fai* and *H. jenkinsii* in BOLD. Four other individuals identified in the field as *H. fai* (# 9–12) and *H. granulata* (# 14) also clustered with this sample and were identified as *H. fai* in GenBank and *H. fai and H. jenkinsii* in BOLD.

The average conspecific genetic distances for *H. fai* (including a sample initially identified as *H. granulata*, #14) and *H. jenkinsii* were 0.03 and 0.4% respectively, while the average genetic distance between *H. fai* and *H. jenkinsii* was ∼ 13%. *H. jenkinsii* showed phylogeographic disjunction between samples from Indonesia/Southeast Asia and Australia, but a small genetic distance of <1%. Another sample, also identified as *H. granulata* (# 13) was clearly divergent in the tree, and matched *H. hortlei* [52] on GenBank (86%) and *H. granulata* on BOLD (99%).

#### Neotrygon kuhlii complex [50]

**Figure 4:**
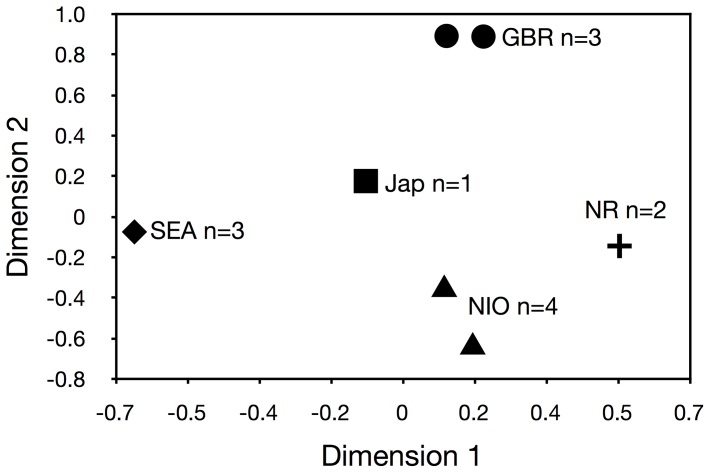
Multidimensional Scaling (MDS) of *Neotrygon kuhlii.* Ningaloo Reef (NR), northern Indian Ocean (NIO), Great Barrier Reef (GBR), Japan (Jap), Southeast Asia (SEA);.

(formerly *Dasyatis kuhlii*). Our sequences of *N. kuhlii* had overall levels of similarity of 99–100% with sequences in both GenBank and BOLD databases. Sequences of unidentified rays from Vietnam (# 66–67) matched closely with *N. kuhlii* (99–100% similarity) on BOLD. The sequences (*n* = 11) provisionally assigned to this species formed five distinct subgroups in the tree and multi-dimensional scaling analysis (Fig. 4) and had an average genetic distance of ∼ 3%. These subgroups were: Great Barrier Reef (# 36–38), Ningaloo Reef (# 39-0), Japan (AB485685.1), northern Indian Ocean (HM467799.1), Indonesia (EU398745.1), and Southeast Asia (Vietnam: # 66–67; Taiwan: EU398735.1). Average distances among and within these groups were 3 and 0.15%, respectively. The most divergent lineage was from the Great Barrier Reef, which had an average genetic difference of 3.5% from the other sequences from this species. While there was generally a correspondence between the genetic distance and geographical proximity, the two Australian lineages from the western and eastern coasts had the greatest genetic distance (3.8%).

#### *Aetobatus* complex: *A. narinari* Euphrasen 1790/*A. ocellatus* Kuhli 1823

Sequences from Ningaloo Reef identified by different researchers in the field as *A. ocellatus* (# 1–2) and from unidentified samples from the Northern Territory (# 64–65), were identified as *A. narinari* using GenBank and as *A. narinari and A. ocellatus* using BOLD. Sequences of the species commonly referred to as the white-spotted eagle ray from the Caribbean region (Cayman Islands, Belize, and South-East Mexico) (Table S3) and sequences from the Indo-Pacific (Hawaii, Japan, Indonesia, and Australia) showed genetic differences ( = 3.4%). The genetic distance of sequences from *A. narinari* within the Indo-Pacific, including sequences of *A. ocellatus* from Australia was low ( = 0.86%). While the name *A. narinari* is consistently applied to what might be a distinct biological entity in the Caribbean, the names *A. narinari* and *A. ocellatus* seemed to be applied interchangeably to a different biological entity that is widespread in the Indo-Pacific.

#### The ‘*uarnak*’ complex: *Himantura uarnak* (Forsskal 1775), *H. leoparda* [16], *H. astra* [53], *H. toshi* (Whitley, 1939)

Samples of this group were identified by several researchers in the field. They were identified as *H. leoparda* (# 19–21), *H. astra* (# 7, 8) and *H. uarnak* (# 22–33) and all (except #19) were grouped together within the tree with an average genetic distance of 0.15% (ignoring the aberrant sequence of *H. uarnak* from India). Comparisons with the BOLD database identified all these sequences as *H. uarnak.* In contrast, identifications from GenBank were either uninformative or misleading and applied only the name of the order (Rajiformes) (Table S2). The aberrant sequence of *H. uarnak* from India had a genetic distance of 12% from this lineage. The BOLD database identified this sequence as *H. uarnak* (similarity of 100%), *H. gerrardi* [54] (similarity of 99%) and *Dasyatis microps* (similarity of 99%). A sequence from an individual collected from Shoal Bay identified as *H. astra* (# 8) matched sequences labelled as *H. toshi* in GenBank (100%) and as *H. toshi* and *H. astra* (98%) in BOLD. This sequence had an average genetic distance of 0.1% with both *H. astra* and *H. toshi*, suggesting these two species may refer to the same biological species.

## Discussion

Our aim to investigate the applicability of DNA barcoding for confirming field-based identifications of rays, was at best, a partial success. Two kinds of problems limited the successful application of DNA barcoding to rays. First, biological and taxonomic issues included: a) the presence of cryptic species, b) species complexes with a number of named species of uncertain or unresolved taxonomic status and c) widespread species with substantial intra-specific geographical variation. The second set of problems involved the limitations associated with the online databases including: a) insufficient numbers of taxonomically verified entries on GenBank and BOLD databases; and b) the presence of lodged sequences with incorrect, duplicated, outdated, inconsistent or unhelpful names (e.g. insufficient taxonomic resolution). Nevertheless, our study has demonstrated the potential power of the DNA Barcoding approach to confirm field identifications, detect misidentifications, and discover cryptic species and species complexes with taxonomic issues.

As with other barcoding studies of rays [6], the COI gene region was effective for their taxonomic identification and delineation. This was particularly the case for species in which the complexity of their colour patterns made identification difficult without the input from an expert taxonomist. The average intra-specific genetic distance within species (0.63%) we obtained was larger than that reported for Australian chondrichthyans (0.37%) by Ward *et al.* [6]. This could have arisen because we increased the geographic extent of sampling for a number of species. In contrast, the average congeneric distance we recorded (7.5%) was similar (7.4%) to that found by Ward *et al.* [6]. Twenty species of rays representing 9 species tagged as part of an ecological project were correctly and consistently identified using BOLD, albeit with some inconsistent nomenclature (Table S2). In a study of marine fish, Zemlak *et al.* [46] suggested that similarity below 96.5% could be used as a rule of thumb for discriminating species. All of these samples had BOLD matches ≥98%, with these levels well within the tolerance range for intra-specific genetic divergence.

DNA Barcoding has also been useful when only parts of an animal are available for identification e.g. [45], [55], [56]. The value of barcoding in this context was confirmed by the identification of ‘unknown’ species from tissues samples obtained from rays in markets near Ha Long Bay, Vietnam (# 66, 67) and from fishers in the Northern Territory (# 64, 65) as belonging to the *N. kuhlii* and *A. narinari* species complexes, respectively. In both cases, the match between our sequences and the BOLD database was ≥99%. Furthermore, as both species groups displayed significant geographic variation, the confidence of identifications was enhanced due to lodgements on data bases of sequences from individuals from a range of geographic localities.

Barcoding has been used successfully to aid in the identification of species with morphological complexity e.g. [12], [57], [58]. In our study we found that not all field identifications were correct or reliable, with a total of nine specimens representing four species identified incorrectly. Field identification was particularly challenging in the *‘uarnak’* complex group due to similarities in colour patterns among species. While the DNA sequences as summarized in the tree indicated clear taxonomic groupings, the fact that identical reference sequences on the BOLD database were labelled with two different names further complicated taxonomic identification. Lastly, one specimen thought to be a possible new species of *Taeniurops* (# 60) based on colour patterns was unambiguously identified from the BOLD database as *Taeniura meyeni* and was genetically identical to other samples of this species from Ningaloo Reef. Another example of ambiguous taxonomy, which limited the value of barcoding for rays, involved the species *P. atrus* and *P. sephen*. The low sequence divergence and the absence of any geographic structure in the relationships among the sequences of *P. atrus* and *P. sephen* indicated that the sequences available online under these different names are most likely the same species. Furthermore, the close relative found in the Red Sea that was originally named as *Pastinachus sephen* was morphologically different from the Indo-Pacific form [11], [59].

The databases were uninformative for two species. *D. parvonigra* (# 3) was identified simply as *Dasyatis sp* in the BOLD database while GenBank matched an entirely different species, *D. fluviorum* to our sequence. *Neotrygon ningalooensis* (# 42–43) represents a new species [47] for which sequences are not yet available in the databases with no matching sequences greater than a similarity of 89%. Overall, these results show that a great deal of care must be taken when using DNA barcoding to confirm field identifications, particularly with groups that have a recent history of nomenclatural changes. When the online search engines gave ambiguous responses to our sequence submissions, the phylogenetic tree and genetic distances analyses proved useful aids to identification.

The misidentification of several species belonging to the genus *Himantura* on the basis of morphology confirms the taxonomic complexity of the genus, which has been continuously reviewed for the last 10 years [11], [16], [53], [60]. The ‘*uarnak’* complex is a group of whip-rays with spotted, ocellated and reticulated dorsal patterns that up until 2008, had 7 valid nominal species [53]. Identification of members of this complex was further complicated due ontogenetic changes in colour patterns that can lead to misidentification of different life-history stages of the same species [16]. We found field identification of species within this group challenging because of the similarities in colour patterns among *H. uarnak, H. leoparda,* and *H. astra*. The clustering of *H. leoparda* as *H. uarnak* in the tree suggests that these two named species represent the same biological species in this study. While the Australian samples are clearly a distinct species, a sequence from India (GenBank accession number EU541309.1) lodged under the same name is genetically quite different (12%) when compared with the rest of *H. uarnak* sequences and may represent a new species more closely related to *H. gerrardi*.

*Himantura fai* and *H. jenkinsii* also proved difficult to distinguish in the field. As discussed above, the sequences we obtained matched both *H. fai* and *H. jenkinsii* in the BOLD database; however, the tree clearly showed that these are distinct species, suggesting that a revision of the names attached to sequences in the BOLD database is required. Sequences assigned to *H. astra* and *H. toshi* also need to be reviewed [53]. The tree suggests there is only one species, but the BOLD database again produced ambiguous results with our sequences being identified as both *H. toshi* and *H. astra* with similarities of 100%.

Confusion in taxonomy was also a problem for the genus *Aetobatus*. *Aetobauts narinari* represents a widespread species complex and the pattern of geographic variation in COI indicates that there are two closely related forms. One distinct species, *A. narinari,* occurs in the north Atlantic and the other that occurs in the Indo-Pacific should be referred to *A. ocellatus* [61], [62]. To add to the uncertainty involving these species, the BOLD database identified our sequences as both *A. narinari* and *A. ocellatus*. Our results were consistent with those of Richards *et al*. [62] and Schluessel *et al.* [63] who analysed sequences of cytochrome b and COI and found that individuals of *A. narinari* from the west Atlantic formed a distinct lineage compared with those from the Indo-Pacific. Based on a morphological review, White *et al*. [61] proposed that *A. ocellatus* is a separate species restricted to the Indo-West Pacific and distinct from the *A. narinari* complex. The average genetic distance between sequences of *A. narinari* from the Caribbean Sea and sequences from the Indo-Pacific region labelled as *A. narinari* in our study was 3.4%, consistent with the idea that the Atlantic and Indo-Pacific lineages are separate species. This pattern and geographical divergence between Atlantic and Pacific stocks has been observed in other elasmobranchs such as *Squalus acanthias* [64].

The *Neotrygon kuhlii* species complex is also widespread, with the maximum divergence close to the rule of thumb for discriminating species. Geographic differences in genetic divergences indicate the possibility of three differentiated clades consisting of a) east Asia (Vietnam, Taiwan, and Japan), b) the eastern Indian Ocean (India, Indonesia, and Ningaloo Reef, Australia) and c) the Great Barrier Reef (Australia). This is consistent with the suggestion by Ward *et al.* [6] of the possibility of cryptic species within *N. kuhlii.* Further research is required to determine geographic boundaries and to examine variation in other genes (e.g. microsatellite loci) to establish if this group is undergoing incipient speciation.

We increased the geographic spread of genetic sampling for several rays in the tropical Indo-Pacific and a number of contrasting patterns have emerged that might be of taxonomic or biological importance. Several species were noteworthy for having little genetic divergence over large (1000s of km) distances. For example, *Glaucostegus typus* (# 4–6) shared haplotypes between Ningaloo Reef, Western Australia and Northern Territory; *U. asperrimus* (# 61 63) shared haplotypes between Ningaloo Reef, Western Australia and Queensland; *H. fai* (# 9–12, 18) and *P. atrus* (# 44–49) both shared haplotypes between Ningaloo Reef, the Northern Territory and Malaysia. These results suggest that these species all have high vagility, at least at generational time scales.

In contrast, *T. lymma* (# 50–53, 56, 57), *H. jenkinsii* (# 15, 16), and *M. alfredi* (# 34, 35) showed little (<1%) but potentially biologically relevant variation in sequences between Australia and Indonesia. While our sample sizes were small, this result implies that the biogeographic factors responsible for population differentiation could potentially act on the three species in a similar way. The possibility of population differentiation in *M. alfredi* is supported by the observations of strong residency patterns in Indonesia [65] and in Ningaloo Reef (F. McGregor, *pers. comm*.) based on acoustic tagging and photo-identification studies. An individual misidentified in the field as *D. parvonigra* (# 3) from the Northern Territory was in fact a new record of *D. fluviorum,* a species that was previously thought to occur only along the eastern coast of Australia [11].

The extent of genetic divergence within several species (*N. kuhlii, N. leylandi)* from the north-west and east of Australia might reflect historical isolation when the land bridge between New Guinea and northern Australia formed during the Holocene and late Pleistocene [66]. A number of marine and coastal species (including elasmobranchs) show this pattern of differentiation caused by vicariant events [14], [15], [67]–[69]. Further investigation of this idea would require intensive sampling of these rays for both nuclear and mitochondrial markers between Torres Strait and the Arafura-Timor Seas to understand the geographic basis for genetic differentiation. It was, however, surprising to find such discordance between genetic differentiation and body size in some rays (e.g. *T. lymma vs M. alfredi*) because it is generally assumed that body size and dispersal capacity are correlated in elasmobranchs [70]. Several genetic studies have found surprisingly strong population structure in sharks and rays considered vagile that might be related to site fidelity in both adults and juveniles or deep water acting as barriers to dispersal [14], [15], [71], [72].

The general limits and pitfalls of DNA barcoding as a stand-alone tool for identifying species and delimiting taxonomic boundaries have been dealt with elsewhere [10], [73], [74]–[76]. However, it is worth reiterating that taxonomic decision-making solely on the basis of a single maternally inherited marker will not identify all biological species. Other studies of rays have found that mtDNA sequences have not been useful for delimiting species boundaries since haplotypes can be shared, particularly between newly evolved species [12]. Conversely, it is possible that some species with higher genetic distances that approach the (arbitrarily defined) species-level thresholds might be able to interbreed. Such rules-of-thumb for genetic distance will vary in their usefulness among gene regions and across taxonomic groups and will inevitably be a “one-way” test for species discrimination [6], [77], [78].

While we found that barcoding for rays was largely successful as an identification tool, there were several limitations. To succeed, barcoding must be able to reference a stable and well-defined taxonomy and have access to a sufficient number of barcodes lodged on databases that have been verified taxonomically [77]. We discovered that several species groups require taxonomic review both to define confidently species boundaries and revise nomenclatures. Furthermore, the continued updating of sequences lodged on GenBank and BOLD is a vital, but a rarely considered issue in the practical application of barcoding. The specimens from which sequences are derived must first be identified by a competent taxonomist. The names assigned to sequences need to be updated on the online genetic data bases when taxonomies are revised and names changed. Fifty-eight percent of our sequences did not matched entries on GenBank and 30% showed ambiguous results on BOLD due to confusing nomenclature (Table S2). For example, in the cases of *H. fai* versus *H. jenkinsii* and *H. astra* versus *H. toshi*, the BOLD search engine showed a 99–100% similarity with both names in each case, thereby invalidating the simple use of BOLD as an identification tool. Furthermore, a number of ray sequences on GenBank were identified only to genus or family level making them uninformative for DNA barcoding-based identification.

### Conclusions

DNA barcoding was successful in validating field identifications and correcting misidentifications of tagged rays at Ningaloo Reef, WA, although application of the technique was somewhat problematic due to the inconsistency and ambiguity of taxonomic information available on the online data bases. Our genetic analyses have resulted in a better understanding of intra-species diversity and biogeographic patterns along the coast of northern Australia and at localities across the Indo-Pacific that will ultimately be useful for delimiting species boundaries, fisheries management and conservation of tropical rays.

In the future, the usefulness of ray barcoding will be directly related to the quantity and geographic representation of sequences, the number of sequences from taxonomically verified specimens, taxonomic revisions of key species complexes and a revision of the taxonomic nomenclature assigned to existing sequences on genetic data bases. With these advances, together with the recent production of COI sequences and taxonomic studies in Australia [6], [11] and Indonesia [79], [80], barcoding for species identification of rays will become far less problematic, at least for this region. Such an approach needs to be extended to areas with high diversity of rays around the world.

## Supporting Information

Table S1 **Specimen collection details for all sequences obtained in this study.**
(DOCX)

Table S2 **Identification of sampled rays using GenBank and BOLD databases.**
(DOCX)

Table S3 **Online sequences used in this study with their GenBank accession numbers.**
(DOCX)
