# Supplementary material for: Identification of Rays through DNA Barcoding: An Application for Ecologists
Source: PLoS One. 2012 Jun 11;7(6):e36479. doi: 10.1371/journal.pone.0036479 (PMC3372520; doi:10.1371/journal.pone.0036479)
Supplement: Table S1 — Specimen collection details for all sequences obtained in this study. (DOCX) [file pone.0036479.s001.docx]

| **New**  **No.** | **Initial ID** | **Locality** | **Latitude** | **Longitude** | **Collector** | **Used for analysis** | **Identified by taxonomist** | **Tagged** | **GenBank accession #** |
| --- | --- | --- | --- | --- | --- | --- | --- | --- | --- |
| 1 | *Aetobatus ocellatus* | Skeleton Bay, Ningaloo | 23° 7'53.21"S | 113°46'14.76"E | FC | Yes | Yes |  | JQ765503 |
| 2 | *Aetobatus ocellatus* | Skeleton Bay, Ningaloo | 23° 7'53.21"S | 113°46'14.76"E | OOS | Yes | Yes | Yes | JQ765504 |
| 3 | *Dasyatis parvonigra* | Shoal Bay, NT | 12°20'49.44"S | 131° 0'51.37"E | FC | Yes | Yes |  | JQ765505 |
| 4 | *Glaucostegus typus* | Skeleton Bay, Ningaloo | 23° 7'53.21"S | 113°46'14.76"E | FC | Yes | No |  | JQ765506 |
| 5 | *Glaucostegus typus* | Mangrove Bay, Ningaloo | 21°58'5.23"S | 113°56'27.02"E | FC | Yes | No | Yes | JQ765507 |
| 6 | *Glaucostegus typus* | Shoal Bay, NT | 12°20'49.44"S | 131° 0'51.37"E | FC | Yes | No |  | JQ765508 |
| 7 | *Himantura astra* | Skeleton Bay, Ningaloo | 23° 7'53.21"S | 113°46'14.76"E | OOS | Yes | No | Yes | JQ765509 |
| 8 | *Himantura astra* | Shoal Bay, NT | 12°20'49.44"S | 131° 0'51.37"E | FC | Yes | Yes |  | JQ765510 |
| 9 | *Himantura fai* | Point Cloates, Ningaloo | 22°42'22.20"S | 113°39'51.95"E | FC | Yes | Yes |  | JQ765511 |
| 10 | *Himantura fai* | Mangrove Bay, Ningaloo | 21°58'5.23"S | 113°56'27.02"E | FC | Yes | Yes |  | JQ765512 |
| 11 | *Himantura fai* | Mangrove Bay, Ningaloo | 21°58'5.23"S | 113°56'27.02"E | FC | Yes | Yes | Yes | JQ815392 |
| 12 | *Himantura fai* | Skeleton Bay, Ningaloo | 23° 7'53.21"S | 113°46'14.76"E | OOS | Yes | No | Yes | JQ765513 |
| 13 | *Himantura granulata* | GBR, Qld | 14°41'27.55"S | 145°27'29.05"E | MM | Yes | Yes |  | JQ765514 |
| 14 | *Himantura granulata* | Ranger Bay, Ningaloo | 21°53'54.39"S | 113°58'59.75"E | OOS | Yes | No |  | JQ765515 |
| 15 | *Himantura jenkensii* | Mangrove Bay, Ningaloo | 21°58'5.23"S | 113°56'27.02"E | FC | Yes | Yes | Yes | JQ765516 |
| 16 | *Himantura jenkensii* | Mangrove Bay, Ningaloo | 21°58'5.23"S | 113°56'27.02"E | FC | Yes | Yes | Yes | JQ765517 |
| 17 | *Himantura jenkensii* | Mangrove Bay, Ningaloo | 21°58'5.23"S | 113°56'27.02"E | FC | No | Yes | Yes | JQ815393 |
| 18 | *Himantura jenkensii* | Shoal Bay, NT | 12°20'49.44"S | 131° 0'51.37"E | FC | Yes | No |  | JQ765518 |
| 19 | *Himantura leoparda* | Skeleton Bay, Ningaloo | 23° 7'53.21"S | 113°46'14.76"E | OOS | No | No |  | JQ929047 |
| 20 | *Himantura leoparda* | Ranger Bay, Ningaloo | 21°53'54.39"S | 113°58'59.75"E | OOS | Yes | No |  | JQ765519 |
| 21 | *Himantura leoparda* | Ranger Bay, Ningaloo | 21°53'54.39"S | 113°58'59.75"E | OOS | Yes | Yes |  | JQ765520 |
| 22 | *Himantura uarnak* | Skeleton Bay, Ningaloo | 23° 7'53.21"S | 113°46'14.76"E | FC | No | Yes | Yes | JQ815394 |
| 23 | *Himantura uarnak* | Skeleton Bay, Ningaloo | 23° 7'53.21"S | 113°46'14.76"E | FC | Yes | Yes | Yes | JQ765521 |
| 24 | *Himantura uarnak* | Mangrove Bay, Ningaloo | 21°58'5.23"S | 113°56'27.02"E | FC | Yes | Yes | Yes | JQ765522 |
| 25 | *Himantura uarnak* | Skeleton Bay, Ningaloo | 23° 7'53.21"S | 113°46'14.76"E | FC | Yes | No | Yes | JQ765523 |
| 26 | *Himantura uarnak* | Skeleton Bay, Ningaloo | 23° 7'53.21"S | 113°46'14.76"E | FC | Yes | Yes |  | JQ765524 |
| 27 | *Himantura uarnak* | Point Cloates, Ningaloo | 22°42'22.20"S | 113°39'51.95"E | FC | Yes | Yes |  | JQ765525 |
| 28 | *Himantura uarnak* | Point Cloates, Ningaloo | 22°42'22.20"S | 113°39'51.95"E | FC | Yes | Yes |  | JQ765526 |
| 29 | *Himantura uarnak* | Stanley's Pool, Ningaloo | 22°59'29.39"S | 113°48'6.04"E | FC | Yes | Yes | Yes | JQ765527 |
| 30 | *Himantura uarnak* | Mangrove Bay, Ningaloo | 21°58'5.23"S | 113°56'27.02"E | FC | Yes | Yes | Yes | JQ765528 |
| 31 | *Himantura uarnak* | Shoal Bay, NT | 12°20'49.44"S | 131° 0'51.37"E | FC | Yes | No |  | JQ765529 |
| 32 | *Himantura uarnak* | Shoal Bay, NT | 12°20'49.44"S | 131° 0'51.37"E | FC | Yes | Yes |  | JQ765530 |
| 33 | *Himantura uarnak* | Meckets Creek, NT | 12°20'23.42"S | 130°57'22.38"E | FC | No | No |  | JQ815395 |
| 34 | *Manta alfredi* | Stanley's Pool, Ningaloo | 22°59'29.39"S | 113°48'6.04"E | FM* | Yes | Yes |  | JQ765531 |
| 35 | *Manta alfredi* | Stanley's Pool, Ningaloo | 22°59'29.39"S | 113°48'6.04"E | FM* | Yes | Yes |  | JQ765532 |
| 36 | *Neotrygon kuhlii* | GBR, Qld | 14°41'27.55"S | 145°27'29.05"E | MM | Yes | No |  | JQ765533 |
| 37 | *Neotrygon kuhlii* | GBR, Qld | 14°41'27.55"S | 145°27'29.05"E | MM | Yes | No |  | JQ765534 |
| 38 | *Neotrygon kuhlii* | GBR, Qld | 14°41'27.55"S | 145°27'29.05"E | MM | Yes | Yes |  | JQ765535 |
| 39 | *Neotrygon kuhlii* | Point Edgar, Ningaloo | 22°34'52.46"S | 113°39'9.36"E | OOS | Yes | No |  | JQ765536 |
| 40 | *Neotrygon kuhlii* | Skeleton Bay, Ningaloo | 23° 7'53.21"S | 113°46'14.76"E | OOS | Yes | No |  | JQ765537 |
| 41 | *Neotrygon leylandi* | 5 Fingers, Ningaloo | 23°11'38.34"S | 113°46'1.45"E | FC | Yes | No |  | JQ765538 |
| 42 | *Neotrygon ningalooensis* | 5 Fingers, Ningaloo | 23°11'38.34"S | 113°46'1.45"E | FC | Yes | Yes |  | JQ765539 |
| 43 | *Neotrygon ningalooensis* | 5 Fingers, Ningaloo | 23°11'38.34"S | 113°46'1.45"E | FC | Yes | No |  | JQ765540 |
| 44 | *Pastinachus atrus* | Mangrove Bay, Ningaloo | 21°58'5.23"S | 113°56'27.02"E | FC | Yes | No | Yes | JQ765541 |
| 45 | *Pastinachus atrus* | Skeleton Bay, Ningaloo | 23° 7'53.21"S | 113°46'14.76"E | FC | Yes | No |  | JQ765542 |
| 46 | *Pastinachus atrus* | Ranger Bay, Ningaloo | 21°53'54.39"S | 113°58'59.75"E | OOS | Yes | No |  | JQ765543 |
| 47 | *Pastinachus atrus* | Ranger Bay, Ningaloo | 21°53'54.39"S | 113°58'59.75"E | OOS | Yes | No |  | JQ765544 |
| 48 | *Pastinachus atrus* | Meckets Creek, NT | 12°20'23.42"S | 130°57'22.38"E | FC | Yes | Yes |  | JQ765545 |
| 49 | *Pastinachus atrus* | Shoal Bay, NT | 12°20'49.44"S | 131° 0'51.37"E | FC | Yes | Yes |  | JQ765546 |
| 50 | *Taeniura lymma* | Mangrove Bay, Ningaloo | 21°58'5.23"S | 113°56'27.02"E | FC | No | No | Yes | JQ929048 |
| 51 | *Taeniura lymma* | Mangrove Bay, Ningaloo | 21°58'5.23"S | 113°56'27.02"E | FC | Yes | No |  | JQ765547 |
| 52 | *Taeniura lymma* | Mangrove Bay, Ningaloo | 21°58'5.23"S | 113°56'27.02"E | FC | Yes | No |  | JQ765548 |
| 53 | *Taeniura lymma* | Skeleton Bay, Ningaloo | 23° 7'53.21"S | 113°46'14.76"E | OOS | Yes | No | Yes | JQ765549 |
| 54 | *Taeniura lymma* | Point Look, Ningaloo | 21°55'37.51"S | 113°53'38.99"E | OOS | No | No |  | JQ825396 |
| 55 | *Taeniura lymma* | Groote Island, NT | 13°39'15.60"S | 136°56'25.49"E | FC | Yes | No |  | JQ765551 |
| 56 | *Taeniura lymma* | Groote Island, NT | 13°39'15.60"S | 136°56'25.49"E | FC | Yes | No |  | JQ765552 |
| 57 | *Taeniura lymma* | Groote Island, NT | 13°39'15.60"S | 136°56'25.49"E | FC | Yes | No |  | JQ765553 |
| 58 | *Taeniurops meyeni* | Point Look, Ningaloo | 21°55'37.51"S | 113°53'38.99"E | OOS | Yes | No |  | JQ765550 |
| 59 | *Taeniurops meyeni* | 5 Fingers, Ningaloo | 23°11'38.34"S | 113°46'1.45"E | FC | Yes | Yes |  | JQ765554 |
| 60 | *Taeniurops spp* | Point Edgar, Ningaloo | 22°34'52.46"S | 113°39'9.36"E | OOS | Yes | No |  | JQ765555 |
| 61 | *Urogymnus asperrimus* | GBR, Qld | 14°41'27.55"S | 145°27'29.05"E | MM | Yes | No |  | JQ765556 |
| 62 | *Urogymnus asperrimus* | Ranger Bay, Ningaloo | 21°53'54.39"S | 113°58'59.75"E | OOS | Yes | No |  | JQ765557 |
| 63 | *Urogymnus asperrimus* | Point Look, Ningaloo | 21°55'37.51"S | 113°53'38.99"E | OOS | Yes | No |  | JQ765558 |
| 64 | *unknown* | Darwin Harbour, NT | 12°24'26.35"S | 130°47'20.82"E | GJ* | Yes | No |  | JQ765559 |
| 65 | *unknown* | Darwin Harbour, NT | 12°24'26.35"S | 130°47'20.82"E | GJ* | Yes | No |  | JQ765560 |
| 66 | *unknown* | Ha Long Bay, Vietnam | 20°45'38.93"N | 106°52'47.17"E | CA | Yes | No |  | JQ765561 |
| 67 | *unknown* | Ha Long Bay, Vietnam | 20°45'38.93"N | 106°52'47.17"E | CA | Yes | No |  | JQ765562 |

Locality: GBR, Great Barrier Reef; NT, Northern Territory; Qld, Queensland

*GJ, Grant Johnson, NT Fisheries

**FM, Frazer McGregor, Murdoch University

***Only those sequences of 519 bp were used in the phylogenetic analysis
