# Supplementary material for: Identification of Rays through DNA Barcoding: An Application for Ecologists
Source: PLoS One. 2012 Jun 11;7(6):e36479. doi: 10.1371/journal.pone.0036479 (PMC3372520; doi:10.1371/journal.pone.0036479)
Supplement: Table S2 — Identification of sampled rays using GenBank and BOLD databases. (DOCX) [file pone.0036479.s002.docx]

|  | Initial Id | Locality | GenBank Id | % | BOLD Id | % | Used for analysis |
| --- | --- | --- | --- | --- | --- | --- | --- |
| 1 | *Aetobatus ocellatus* Kuhli, 1823 | Skeleton Bay, Ningaloo | *A. narinari* | 100 | *A. narinari/ocellatus* | 100 | Yes |
| 2 | *Aetobatus ocellatus* Kuhli, 1823* | Skeleton Bay, Ningaloo | *A. narinari* | 99 | *A. narinari/ocellatus* | 100 | Yes |
| 3 | *Dasyatis parvonigra,*Last and White, 2008 | Shoal Bay, NT | *D. fluviorum* | 98 | *Dasyiatis sp* | 99 | Yes |
| 4 | *Glaucostegus typus* (Bennett, 1830) | Skeleton Bay, Ningaloo | *G. typus* | 100 | *G. typus/ R. typus* | 100 | Yes |
| 5 | *Glaucostegus typus* (Bennett, 1830)* | Mangrove Bay, Ningaloo | *G. typus* | 99 | *G. typus/ R. typus* | 100 | Yes |
| 6 | *Glaucostegus typus* (Bennett, 1830) | Shoal Bay, NT | *G. typus* | 100 | *G. typus/ R. typus* | 100 | Yes |
| 7 | *Himantura astra,* Last et al., 2008* | Skeleton Bay, Ningaloo | *Rajiformes sp* | 100 | *H. uarnak* | 100 | Yes |
| 8 | *Himantura astra,* Last et al., 2008 | Shoal Bay, NT | *H. toshi* | 100 | *H. toshi/ H. astra* | 98 | Yes |
| 9 | *Himantura fai,* Jordan & Seale, 1906 | Point Cloates, Ningaloo | *H. fai* | 99 | *H. jenkinsii/fai* | 99 | Yes |
| 10 | *Himantura fai,* Jordan & Seale, 1906 | Mangrove Bay, Ningaloo | *H. fai* | 99 | *H. jenkinsii/fai* | 99 | Yes |
| 11 | *Himantura fai,* Jordan & Seale, 1906* | Mangrove Bay, Ningaloo | *H. fai* | 99 | *H. jenkinsii/fai* | 99 | Yes |
| 12 | *Himantura fai,* Jordan & Seale, 1906* | Skeleton Bay, Ningaloo | *H. fai* | 99 | *H. jenkinsii/fai* | 99 | Yes |
| 13 | *Himantura granulata* (Macleay, 1883) | GBR, Qld | *H. hortlei* | 86 | *H. granulata* | 99 | Yes |
| 14 | *Himantura granulata* (Macleay, 1883) | Ranger Bay, Ningaloo | *H. fai* | 100 | *H. jenkinsii/fai* | 100 | Yes |
| 15 | *Himantura jenkinsii* (Annandale, 1990)* | Mangrove Bay, Ningaloo | *H. jenkinsii* | 100 | *H. jenkinsii/fai* | 100 | Yes |
| 16 | *Himantura jenkinsii* (Annandale, 1990) * | Mangrove Bay, Ningaloo | *H. jenkinsii* | 99 | *H. jenkinsii* | 100 | Yes |
| 17 | *Himantura jenkinsii* (Annandale, 1990) * | Mangrove Bay, Ningaloo | *H. jenkinsii* | 100 | *H. jenkinsii* | 100 | No |
| 18 | *Himantura jenkinsii* (Annandale, 1990) | Shoal Bay, NT | *H. fai* | 100 | *H. jenkinsii/fai* | 100 | Yes |
| 19 | *Himantura leoparda* Manjaji-Matsumoto and Last, 2008 | Skeleton Bay, Ningaloo | *Rajiformes sp* | 99 | *H. uarnak* | 100 | No |
| 20 | *Himantura leoparda* Manjaji-Matsumoto and Last, 2008 | Ranger Bay, Ningaloo | *Rajiformes sp* | 100 | *H. uarnak* | 100 | Yes |
| 21 | *Himantura leoparda* Manjaji-Matsumoto and Last, 2008 | Ranger Bay, Ningaloo | *Rajiformes sp* | 100 | *H. uarnak* | 100 | Yes |
| 22 | *Himantura uarnak* (Forskal, 1775)* | Skeleton Bay, Ningaloo | *Rajiformes sp* | 99 | *H. uarnak* | 100 | No |
| 23 | *Himantura uarnak* (Forskal, 1775)* | Skeleton Bay, Ningaloo | *Rajiformes sp* | 100 | *H. uarnak* | 100 | Yes |
| 24 | *Himantura uarnak* (Forskal, 1775)* | Mangrove Bay, Ningaloo | *Rajiformes sp* | 100 | *H. uarnak* | 100 | Yes |
| 25 | *Himantura uarnak* (Forskal, 1775)* | Skeleton Bay, Ningaloo | *Rajiformes sp* | 100 | *H. uarnak* | 100 | Yes |
| 26 | *Himantura uarnak* (Forskal, 1775) | Skeleton Bay, Ningaloo | *Rajiformes sp* | 99 | *H. uarnak* | 100 | Yes |
| 27 | *Himantura uarnak* (Forskal, 1775) | Point Cloates, Ningaloo | *Rajiformes sp* | 99 | *H. uarnak* | 99 | Yes |
| 28 | *Himantura uarnak* (Forskal, 1775) | Point Cloates, Ningaloo | *Rajiformes sp* | 99 | *H. uarnak* | 99 | Yes |
| 29 | *Himantura uarnak* (Forskal, 1775)* | Stanley's Pool, Ningaloo | *Rajiformes sp* | 100 | *H. uarnak* | 100 | Yes |
| 30 | *Himantura uarnak* (Forskal, 1775)* | Mangrove Bay, Ningaloo | *Rajiformes sp* | 98 | *H. uarnak* | 100 | Yes |
| 31 | *Himantura uarnak* (Forskal, 1775) | Shoal Bay, NT | *Rajiformes sp* | 100 | *H. uarnak* | 100 | Yes |
| 32 | *Himantura uarnak* (Forskal, 1775) | Shoal Bay, NT | *Rajiformes sp* | 100 | *H. uarnak* | 100 | Yes |
| 33 | *Himantura uarnak* (Forskal, 1775) | Meckets Creek, NT | *Rajiformes sp* | 95 | *H. uarnak* | 100 | No |
| 34 | *Manta alfredi* (Krefft, 1868) | Stanley's Pool, Ningaloo | *M. birostris* | 99 | *M .birostris* | 99 | Yes |
| 35 | *Manta alfredi* (Krefft, 1868) | Stanley's Pool, Ningaloo | *M. birostris* | 99 | *M .birostris* | 99 | Yes |
| 36 | *Neotrygon kuhlii* (Last and White, 2008) | GBR, Qld | *Rajiformes sp* | 99 | *N. kuhlii* | 100 | Yes |
|  |  |  | *N. kuhlii* | 96 |  |  |  |
| 37 | *Neotrygon kuhlii* (Last and White, 2008) | GBR, Qld | *Rajiformes sp* | 99 | *N. kuhlii* | 100 | Yes |
|  |  |  | *N. kuhlii* | 96 |  |  |  |
| 38 | *Neotrygon kuhlii* (Last and White, 2008) | GBR, Qld | *Rajiformes sp* | 99 | *N. kuhlii* | 99 | Yes |
|  |  |  | *N. kuhlii* | 96 |  |  |  |
| 39 | *Neotrygon kuhlii* (Last and White, 2008) | Point Edgar, Ningaloo | *Rajiformes sp* | 99 | *N. kuhlii* | 99 | Yes |
|  |  |  | *N. kuhlii* | 96 |  |  |  |
| 40 | *Neotrygon kuhlii* (Last and White, 2008) | Skeleton Bay, Ningaloo | *Rajiformes sp* | 99 | *N. kuhlii* | 99 | Yes |
|  |  |  | *N. kuhlii* | 96 |  |  |  |
| 41 | *Neotrygon leylandi* (Last, 1987) | 5 Fingers, Ningaloo | *D. leylandi* | 99 | *N. leylandi* | 100 | Yes |
| 42 | *Neotrygon ningalooensis* (Last et al., 2010) | 5 Fingers, Ningaloo | *Rajiformes sp* | 94 | *unable to match* |  | Yes |
| 43 | *Neotrygon ningalooensis* (Last et al., 2010) | 5 Fingers, Ningaloo | *Rajiformes sp* | 93 | *unable to match* |  | Yes |
| 44 | *Pastinachus atrus* (Macleay, 1993)* | Mangrove Bay, Ningaloo | *P. sephen* | 100 | *P. atrus/sephen* | 99 | Yes |
| 45 | *Pastinachus atrus* (Macleay, 1993) | Skeleton Bay, Ningaloo | *P. sephen* | 99 | *P. atrus/sephen* | 100 | Yes |
| 46 | *Pastinachus atrus* (Macleay, 1993) | Ranger Bay, Ningaloo | *P. sephen* | 100 | *P. atrus/sephen* | 100 | Yes |
| 47 | *Pastinachus atrus* (Macleay, 1993) | Ranger Bay, Ningaloo | *P. sephen* | 100 | *P. atrus/sephen* | 100 | Yes |
| 48 | *Pastinachus atrus* (Macleay, 1993) | Meckets Creek, NT | *P. sephen* | 99 | *P. atrus/sephen* | 94 | Yes |
| 49 | *Pastinachus atrus* (Macleay, 1993) | Shoal Bay, NT | *P. sephen* | 99 | *P. atrus/sephen* | 99 | Yes |
| 50 | *Taeniura lymma* (Forsskal 1775)* | Mangrove Bay, Ningaloo | *T. lymma* | 98 | *T. lymma* | 99 | No |
| 51 | *Taeniura lymma* (Forsskal 1775) | Mangrove Bay, Ningaloo | *T. lymma* | 99 | *T. lymma* | 100 | Yes |
| 52 | *Taeniura lymma* (Forsskal 1775) | Mangrove Bay, Ningaloo | *T. lymma* | 99 | *T. lymma* | 100 | Yes |
| 53 | *Taeniura lymma* (Forsskal 1775)* | Skeleton Bay, Ningaloo | *T. lymma* | 99 | *T. lymma* | 100 | Yes |
| 54 | *Taeniura lymma* (Forsskal 1775) | Point Look, Ningaloo | *T. lymma* | 99 | *T. lymma* | 99 | No |
| 55 | *Taeniura lymma* (Forsskal 1775) | Groote Island, NT | *T. lymma* | 99 | *T. lymma* | 100 | Yes |
| 56 | *Taeniura lymma* (Forsskal 1775) | Groote Island, NT | *T. lymma* | 99 | *T. lymma* | 100 | Yes |
| 57 | *Taeniura lymma* (Forsskal 1775) | Groote Island, NT | *T. lymma* | 99 | *T. lymma* | 98 | Yes |
| 58 | *Taeniurops meyeni* (Muller & Henle, 1841) | Point Look, Ningaloo | *Taeniura meyeni* | 98 | *Taeniura meyeni* | 100 | Yes |
| 59 | *Taeniurops meyeni* (Muller & Henle, 1841) | 5 Fingers, Ningaloo | *Taeniura meyeni* | 98 | *Taeniura meyeni* | 100 | Yes |
| 60 | *Taeniurops spp* | Ranger Bay, Ningaloo | *Taeniura meyeni* | 98 | *Taeniura meyeni* | 100 | Yes |
| 61 | *Urogymnus asperrimus* (Bloch & Scneider, 1801) | GBR, Qld | *Rajiformes sp* | 88 | *U. asperrimus* | 100 | Yes |
| 62 | *Urogymnus asperrimus* (Bloch & Scneider, 1801) | Ranger Bay, Ningaloo | *Rajiformes sp* | 88 | *U. asperrimus* | 99 | Yes |
| 63 | *Urogymnus asperrimus* (Bloch & Scneider, 1801) | Point Look, Ningaloo | *Rajiformes sp* | 88 | *U. asperrimus* | 98 | Yes |
| 64 | *unknown* | Darwin Harbour, NT | *A. narinari* | 98 | *A. narinari/ocellatus* | 99 | Yes |
| 65 | *unknown* | Darwin Harbour, NT | *A. narinari* | 98 | *A. narinari/ocellatus* | 99 | Yes |
| 66 | *unknown* | Ha Long Bay, Vietnam | *N. kuhlii* | 99 | *N. kuhlii* | 99 | Yes |
| 67 | *unknown* | Ha Long Bay, Vietnam | *N. kuhlii* | 100 | *N. kuhlii* | 100 | Yes |

*Tagged ray
