# Supplementary material for: Identification of Rays through DNA Barcoding: An Application for Ecologists
Source: PLoS One. 2012 Jun 11;7(6):e36479. doi: 10.1371/journal.pone.0036479 (PMC3372520; doi:10.1371/journal.pone.0036479)
Supplement: Table S3 — Online sequences used in this study with their GenBank accession numbers. (DOCX) [file pone.0036479.s003.docx]

| No. | Species | Locality | GenBank No. | Author |
| --- | --- | --- | --- | --- |
| 1 | *Aetobatus narinari* | Cayman Islands, Caribbean | FJ812203.1 | Richards *et al*, 2009 |
| 2 | *Aetobatus narinari* | Belize | FJ812202.1 | Richards *et al*, 2009 |
| 3 | *Aetobatus narinari* | Mexico | FJ812200.1 | Richards *et al*, 2009 |
| 4 | *Aetobatus narinari* | Indonesia | FJ812198.1 | Richards *et al*, 2009 |
| 5 | *Aetobatus narinari* | Japan | FJ812196.1 | Richards *et al*, 2009 |
| 6 | *Aetobatus narinari* | Hawaii | FJ812197.1 | Richards *et al*, 2009 |
| 7 | *Aetobatus ocellatus* | Northern WA, Au | EU398507.1 | Ward *et al*, 2008 |
| 8 | *Aetobatus ocellatus* | Lombok, Indonesia | EU398508.1 | Ward *et al,* 2008 |
| 9 | *Carcharhius amblyrhynchos* | Indonesia | EF609308.1 | Ward & Holmes 2007 |
| 10 | *Carcharhius plumbeus* | Lombok, Indonesia | EU398639.1 | Ward *et al*, 2008 |
| 11 | *Dasyatis fluviorum* | Logan River, Qld, Au | DQ108183.1 | Ward *et al*, 2005 |
| 12 | *Dasyatis leylandi* | North-West Shelf, WA, Au | EU398751.1 | Ward *et al*, 2008 |
| 13 | *Dasyatis leylandi* | North-West Shelf, WA, Au | EU398750.1 | Ward *et al*, 2008 |
| 14 | *Dasyatis leylandi* | North-West Shelf, WA, Au | EU398749.1 | Ward *et al*, 2008 |
| 15 | *Dasyatis leylandi* | Rockhampton, Qld, Au | DQ108175.1 | Ward *et al*, 2008 |
| 16 | *Dasyatis leylandi* | Northern Qld, Au | DQ108174.1 | Ward *et al*, 2008 |
| 17 | *Dasyatis parvonigra* | Bali, Indonesia | EU398732.1 | Ward *et al*, 2008 |
| 18 | *Glaucostegus typus* | Northern Qld, Au | EU398999.1 | Ward *et al*, 2008 |
| 19 | *Himantura astra* | Shark Bay, WA, Au | EU398866.1 | Ward *et al*, 2008 |
| 20 | *Himantura astra* | Shelburne Bay, Qld, Au | DQ108170.1 | Ward *et al*, 2008 |
| 21 | *Himantura fai* | Lombok, Indonesia | EU398839.1 | Ward *et al*, 2008 |
| 22 | *Himantura fai* | Sandakan, Malaysia | DQ108176.1 | Ward *et al*, 2008 |
| 23 | *Himantura fava* | Sandakan, Malaysia | DQ108167.1 | Ward *et al*, 2008 |
| 24 | *Himantura gerrardi* | Jakarta, Indonesia | EU398844.1 | Ward *et al*, 2008 |
| 25 | *Himantura gerrardi* | Jakarta, Indonesia | EU398842.1 | Ward *et al*, 2008 |
| 26 | *Himantura hortlei* | Java, Indonesia | EU398848.1 | Ward *et al*, 2008 |
| 27 | *Himantura jenkinsii* | Java, Indonesia | EU398851.1 | Ward *et al*, 2008 |
| 28 | *Himantura jenkinsii* | Legendre Island, WA, Au | DQ108169.1 | Ward *et al*, 2008 |
| 29 | *Himantura jenkinsii* | Sandakan, Malaysia | DQ108168.1 | Ward *et al*, 2008 |
| 30 | *Himantura pastinacoides* | Sarawak, Malaysia | EU398857.1 | Ward *et al*, 2008 |
| 31 | *Himantura pastinacoides* | Kampung, Malaysia | EU398856.1 | Ward *et al*, 2008 |
| 32 | *Himantura toshi* | Hervey Bay, Qld, Au | EU398869.1 | Ward *et al*, 2008 |
| 33 | *Himantura uarnak* | India | EU541309.1 | Persis *et al,* 2008 |
| 34 | *Himantura walga* | Jakarta, Indonesia | EU398876.1 | Ward *et al,* 2008 |
| 35 | *Himantura walga* | Jakarta, Indonesia | EU398874.1 | Ward *et al,* 2008 |
| 36 | *Manta birostris* | Lombok, Indonesia | EU398904.1 | Ward *et al,* 2008 |
| 37 | *Manta birostris* | Indonesia | GU673824.1 | Ward, Last, White pers. com. |
| 38 | *Neotrygon kuhlii* | Japan | AB485685.1 | Yagishita *et al,* 2009 |
| 39 | *Neotrygon kuhlii* | India | HM467799.1 | Bineesh *et al,* 2010 |
| 40 | *Neotrygon kuhlii* | Indonesia | EU398745.1 | Ward & Holmes 2007 |
| 41 | *Neotrygon kuhlii* | Taiwan | EU398735.1 | Ward *et al,* 2008 |
| 42 | *Pastinachus sephen* | Malaysia | EU398973.1 | Ward *et al,* 2008 |
| 43 | *Pastinachus sephen* | Malaysia | EU398972.1 | Ward *et al,* 2008 |
| 44 | *Pastinachus sephen* | Indonesia | EU398971.1 | Ward *et al*, 2008 |
| 45 | *Pastinachus sephen* | Merauke, Indonesia | EU398970.1 | Ward *et al,* 2005 |
| 46 | *Pastinachus solocirostris* | Malaysia | EF609431.1 | Ward & Holmes 2007 |
| 47 | *Rajiformes (H. uarnak)* | WA, Au | GU673418.1 | Ward, Last, White pers.com. |
| 48 | *Rajiformes (H. uarnak)* | WA, Au | GU673414.1 | Ward, Last, White pers.com. |
| 49 | *Rajiformes (H. uarnak)* | WA, Au | GU673417.1 | Ward, Last, White pers.com. |
| 50 | *Rajiformes (P. sephen)* | WA, Au | GU673072.1 | Ward, Last, White pers.com. |
| 51 | *Rajiformes (T. meyeni)* | Qld, Au | GU673424.1 | Ward, Last, White pers.com. |
| 52 | *Taeniura lymma* | Indonesia | FJ584168.1 | Steinke *et al,* 2009 |
| 53 | *Taeniurops meyeni* | India | HM467797.1 | Bineesh *et al*, 2010 |

Locality: GBR, Great Barrier Reef; NT, Northern Territory; Qld, Queensland
